# Comment on “circSMARCA5 Functions as a Diagnostic and Prognostic Biomarker for Gastric Cancer”

**DOI:** 10.1155/2019/2790379

**Published:** 2019-09-12

**Authors:** Farid Rahimi, Amin Talebi Bezmin Abadi

**Affiliations:** ^1^Research School of Biology, The Australian National University, Canberra, ACT 2600, Australia; ^2^Department of Bacteriology, Faculty of Medical Sciences, Tarbiat Modares University, Tehran, Iran

Presently, multiple potential biomarkers have been introduced following many prospective studies [1, 2]. However, none (at least 99%) had been validated according to the rigorous and critical clinical requirements [3, 4]. We read with great interest the paper by Cai et al. titled “circSMARCA5 Functions as a Diagnostic and Prognostic Biomarker for Gastric Cancer,” published in a recent issue of Disease Markers [1] and positing that circSMARCA5 can potentially act as a prognostic and diagnostic biomarker of gastric cancer. We would like to highlight that the study, like any, has certain limitations, including sample size and population bias, which the authors identified but must be interrogated in future studies on biomarkers in gastric cancer patients. More importantly, we would like to pinpoint some important flaws in the design, implementation, and conclusions of the study by Cai et al. Firstly, Cai et al. used tissue and blood samples from patients who underwent radical surgery [1]. circSMARCA5 was measured also in plasma samples. The authors reported a downregulation of circSMARCA5 in gastric cancer patients' tissues and plasma, an observation that discords with the upregulation of the same circular RNA reported in prostate cancer [5–7]. Such discrepancies regarding biomarkers in different cancers may indicate either different mechanistic factors—which are difficult to decipher and reconcile—or more likely discrepant methodological approaches, leading to opposite conclusions about the same biomarker in different cancers. Gastric cancer is a deadly disease, and taking biopsy samples from such patients for experimental or potentially future clinical assessment of biomarkers likely generates an unjustifiable hazard and stress to patients. Secondly, such studies should be validated after establishing (with ethical considerations) the primary experiments using samples from healthy individuals who had been through the same long-term follow-up to mirror the follow-up and the course of the disease in patients. We believe that this overlooked point is the main reason that an ideal biomarker is lacking for the global management of gastric cancer. Thirdly, some technical aspects of the work are questionable; for example, the rationale behind using GAPDH as a control for circular RNAs after the treatment of all the linear RNA pool by RNase R is difficult to understand while GAPDH represents linear RNA, and RNase R degrades linear RNA. A better way of controlling for differences in the expression of circular RNAs would be to use the ΔCT method underlying quantitative reverse-transcriptase PCR [8]. Furthermore, the use of GAPDH as a quantifying guide and control gene in cancer is inherently unreliable because its expression is likely deregulated in various cancer cells [9].

Given the inevitably valuable role of biomarkers in the diagnosis of gastric cancer and their role in developing personalized medicine, clinicians strive to continue studying potential candidates; thus, identifying pitfalls before designing biomarker studies is valuable for patient participants and outcomes of such studies. Finally, the investigation of the mechanisms underlying the involvement of circular RNAs in the development of gastric cancer in patients of diverse ethnicities is another challenging but worthwhile undertaking—which is also lacking in the field.

## References

[1] Cai J., Chen Z., Zuo X. (2019). circSMARCA5 functions as a diagnostic and prognostic biomarker for gastric cancer. *Disease Markers*.

[2] Baratieh Z., Khalaj Z., Honardoost M. A. (2017). Aberrant expression of PlncRNA-1 and TUG1: potential biomarkers for gastric cancer diagnosis and clinically monitoring cancer progression. *Biomarkers in Medicine*.

[3] Qi P., Zhou X., Du X. (2016). Circulating long non-coding RNAs in cancer: current status and future perspectives. *Molecular Cancer*.

[4] Faruq O., Vecchione A. (2015). MicroRNA: diagnostic perspective. *Frontiers in Medicine*.

[5] Matsuoka T., Yashiro M. (2018). Biomarkers of gastric cancer: current topics and future perspective. *World Journal of Gastroenterology*.

[6] Yu J., Xu Q. G., Wang Z. G. (2018). Circular RNA cSMARCA5 inhibits growth and metastasis in hepatocellular carcinoma. *Journal of Hepatology*.

[7] Zhang G., Sun W., Zhu L., Feng Y., Wu L., Li T. (2019). Overexpressed circ_0029426 in glioblastoma forecasts unfavorable prognosis and promotes cell progression by sponging miR-197. *Journal of Cellular Biochemistry*.

[8] Panda A. C., Gorospe M. (2018). Detection and analysis of circular RNAs by RT-PCR. *Bio-Protocol*.

[9] Zhang J.-Y., Zhang F., Hong C.-Q. (2015). Critical protein GAPDH and its regulatory mechanisms in cancer cells. *Cancer Biology & Medicine*.

